# Slipped Capital Femoral Epiphysis in Two Neutered Male Maine Coon Cats: A Case Report

**DOI:** 10.3390/ani16213327

**Published:** 2026-10-22

**Authors:** Cristina Vaduva, Cristian Zaha, Tudor Casalean, Doru Morar, Florin Simiz, Velescu Simina, Florea Bogdan, Corina Marina Kracunovic, Janos Degi, Roxana Dascălu

**Affiliations:** Horia Cernescu Research Unit, Faculty of Veterinary Medicine, University of Life Sciences “Regele Mihai I” from Timisoara, 300645 Timisoara, Romania; cristina.vaduva@usvt.ro (C.V.); tudor.casalean@usvt.ro (T.C.); dorumorar@usvt.ro (D.M.); florinsimiz@usvt.ro (F.S.); simina.velescu@usvt.ro (V.S.); bogdan.florea@usvt.ro (F.B.); corina.kracunovic@usvt.ro (C.M.K.); janosdegi@usvt.ro (J.D.); roxanadascalu@usvt.ro (R.D.)

**Keywords:** delayed physeal closure, coxofemoral joint, diagnostic imaging, feline orthopedics

## Abstract

Slipped capital femoral epiphysis (SCFE), also referred to as spontaneous femoral capital physeal fracture, is an uncommon orthopedic disorder in cats characterized by separation of the femoral capital epiphysis through the proximal femoral physis in the absence of significant trauma. Maine Coon cats, particularly males, appear to be predisposed to this condition. This report describes two neutered male Maine Coon cats, aged 21 and 16 months, presenting with pelvic limb lameness without a known history of significant trauma. The first cat exhibited grade 2 pelvic limb lameness, bilateral pelvic limb muscle atrophy, and pain and crepitus during manipulation of both coxofemoral joints. Radiographic examination revealed bilateral SCFE associated with secondary degenerative changes. The second cat presented with grade 1 pelvic limb lameness, joint laxity, and pain without palpable crepitus. Radiographic examination raised suspicion of right femoral capital physeal separation, which was subsequently confirmed by computed tomography. Both cats underwent femoral head and neck excision (FHNE), with bilateral FHNE performed during a single anesthetic episode in the first cat. Postoperative clinical examinations and owner reports indicated improvement in pelvic limb function and resolution of clinically apparent lameness in both cats. Radiographic examinations performed six months postoperatively documented the surgical changes, with persistent reduction in pelvic limb soft-tissue volume consistent with muscle atrophy. These cases highlight the variable clinical and imaging presentations of SCFE in Maine Coon cats and the complementary roles of radiography and computed tomography in diagnosis. Although clinical improvement was observed following FHNE, the absence of standardized functional assessments limits definitive conclusions regarding the extent of postoperative recovery.

## Simple Summary

Slipped capital femoral epiphysis (SCFE) is an uncommon orthopedic condition in cats characterized by separation of the femoral head through the proximal femoral growth plate, often without a history of significant trauma. Maine Coon cats, particularly males, appear to be predisposed to this condition. This report describes two neutered male Maine Coon cats, aged 21 and 16 months, presenting with pelvic limb lameness. The first cat exhibited bilateral SCFE associated with secondary degenerative joint changes, whereas the second cat presented with unilateral SCFE. Diagnosis was established by radiography in both cases, with computed tomography providing additional diagnostic information in the second case. Both cats underwent femoral head and neck excision (FHNE), with bilateral surgery performed during a single anesthetic episode in the first cat. Postoperative clinical observations and owner reports indicated improvement in pelvic limb function and resolution of clinically apparent lameness in both cats. Follow-up radiographs obtained six months after surgery documented the surgical changes, although muscle atrophy remained evident. These cases highlight the importance of considering SCFE in young Maine Coon cats presenting with pelvic limb lameness, even in the absence of significant trauma.

## 1. Introduction

Femoral capital physeal fractures are uncommon orthopedic conditions in cats, characterized by separation of the femoral head from the femoral neck through the proximal femoral physis [1,2]. When this separation occurs spontaneously or in the absence of significant trauma, the condition is commonly referred to as slipped capital femoral epiphysis (SCFE). SCFE has been reported predominantly in young male cats, particularly neutered males, and has been associated with abnormalities or persistence of the proximal femoral physis [3,4].

Several factors have been proposed to contribute to the development of this condition, including sex, body weight, skeletal maturation, breed, and neutering status [3,4]. Among the breeds affected, Maine Coon cats appear to be particularly predisposed to SCFE, with a higher reported occurrence compared with the general feline population [3]. This breed-associated predisposition raises the possibility of an underlying hereditary or genetic contribution. However, specific genetic variants associated with SCFE in Maine Coon cats have not been identified, and a hereditary basis has not been established. Therefore, the increased occurrence of SCFE in this breed may reflect a combination of breed-related skeletal characteristics, mechanical loading, and individual variation in physeal maturation rather than a confirmed genetic disorder [3].

Gonadal hormones play an important role in skeletal maturation and physeal closure, and gonadectomy has been associated with delayed closure of some growth plates in cats [3,5]. However, a direct effect of neutering on closure of the proximal femoral physis has not been clearly established. This relationship may be particularly relevant in large, slow-maturing breeds such as the Maine Coon, in which prolonged skeletal maturation and delayed physeal closure have been reported [3,5]. Persistence of an open physis may prolong the period during which the proximal femoral region remains mechanically vulnerable, potentially contributing to physeal failure and development of SCFE under normal or relatively low mechanical loads [4,6].

Clinical presentation of SCFE may vary from progressive or intermittent pelvic limb lameness to an acute onset of severe lameness, frequently without a history of significant trauma [3,7]. Affected cats may exhibit reduced weight-bearing, reluctance to jump, decreased mobility, pain during manipulation of the coxofemoral joint, and reduced range of motion. In Maine Coon cats with SCFE, clinical signs have been reported to develop over periods ranging from a few days to several weeks before presentation [3,6]. Progressive weakening of a persistent or abnormal proximal femoral physis may eventually result in displacement or complete separation of the femoral capital epiphysis, potentially explaining the sudden onset or acute worsening of lameness observed in some affected cats [3,4].

Diagnosis is based on clinical and orthopedic examination combined with diagnostic imaging [2]. Infrared thermography has also been investigated as a non-invasive complementary method for the assessment of painful and musculoskeletal conditions in small animals [8,9]. Previous studies in cats have demonstrated its potential for identifying potentially painful regions, while studies in dogs have reported thermographic alterations associated with orthopedic and neurological disorders [8,9]. However, thermography should be considered an adjunctive method and does not replace structural imaging for definitive orthopedic diagnosis. Radiography is generally the first-line imaging modality and may reveal widening or irregularity of the proximal femoral physis, osteolysis and sclerosis of the femoral neck, metaphyseal remodeling, and variable displacement of the femoral capital epiphysis [1,10].

Ventrodorsal extended-leg and frog-leg projections may be used, with the latter being particularly useful for detecting subtle physeal widening or displacement [2,11]. When radiographic findings are inconclusive or a more detailed assessment of the proximal femur is required, computed tomography (CT) may provide additional information regarding the location, configuration, and extent of the lesion [12].

Gonadal hormones play an important role in skeletal maturation and physeal closure [13]. Estrogens, including those produced through the aromatization of testosterone in males, contribute to the regulation of growth plate maturation and eventual physeal fusion. Consequently, gonadectomy performed before skeletal maturity may delay physeal closure by reducing circulating sex hormone concentrations, potentially prolonging the period during which the growth plate remains mechanically vulnerable [13]. In cats, delayed physeal closure following neutering has been documented; however, a direct causal relationship between gonadectomy and the development of SCFE has not been established [13].

This report describes two male Maine Coon cats, both kept exclusively indoors, that developed pelvic limb lameness associated with femoral capital physeal fractures consistent with SCFE, without a known history of significant trauma. Both cats had been neutered before fracture diagnosis. The diagnosis was established by radiographic examination in Case 1 and CT in Case 2. The aim of this report is to describe the clinical presentation, diagnostic imaging findings, surgical management, and outcome of these two cases and to discuss the potential contribution of prolonged skeletal maturation, persistent physeal activity, and neutering status to the development of SCFE in young male Maine Coon cats.

## 2. Method: Case Presentation

### 2.1. Case 1

A 21-month-old castrated male Maine Coon cat was presented with pelvic limb lameness. The cat was kept exclusively indoors and had a relatively sedentary lifestyle, with no history of significant trauma reported by the owner. Elective neutering had been performed at 8 months of age, when the cat weighed 4.6 kg. Approximately 3 months after neutering, the owner noticed a progressive reduction in physical activity, with the cat preferring to rest rather than walk or engage in its usual activities. A gradual increase in body weight was also observed during this period. Approximately 1 year after neutering, the owner noticed pelvic limb lameness, particularly when the cat stood up after periods of recumbency, prompting veterinary examination. At the time of presentation, the cat weighed 7.8 kg.

At the time of clinical presentation, the cat had a body condition score (BCS) of 7/9, assessed using the nine-point scoring system [13]. Orthopedic examination revealed grade 2 pelvic limb lameness and bilateral pelvic limb muscle atrophy [14]. Palpation and manipulation of both coxofemoral joints elicited pain and revealed crepitus. Based on the clinical history and orthopedic findings, bilateral coxofemoral joint disease was suspected.

Radiographic examination of the pelvis was performed in a ventrodorsal projection under sedation with medetomidine (0.02 mg/kg; Domitor^®^, Orion Corporation, Espoo, Finland), butorphanol (0.2 mg/kg; Butomidor^®^, Richter Pharma AG, Wels, Austria), and ketamine (3 mg/kg; Narkamon^®^, Bioveta a.s., Ivanovice na Hané, Czech Republic), with the cat positioned in dorsal recumbency and both pelvic limbs extended and positioned symmetrically. Radiographic examination revealed bilateral separation and displacement of the femoral capital epiphyses from the femoral necks, consistent with slipped capital femoral epiphysis (SCFE) (Figure 1a). The right proximal femur exhibited more pronounced abnormalities, including irregularity and remodeling of the femoral neck, whereas the left proximal femur showed less extensive physeal separation and displacement. Periarticular remodeling and degenerative changes consistent with secondary osteoarthritis were also evident. Based on the clinical and radiographic findings, bilateral lesions consistent with SCFE and secondary degenerative joint disease were identified, and surgical treatment was recommended. In Case 1, bilateral FHNE was performed during a single anesthetic episode, with both coxofemoral joints treated sequentially during the same surgical intervention.

At the 6-month postoperative follow-up, radiographic examination demonstrated bilateral removal of the femoral heads and necks without apparent osseous contact between the proximal femora and the acetabula. Bilateral reduction in pelvic limb soft-tissue volume, consistent with muscle atrophy, remained evident on the follow-up radiograph (Figure 1b).

### 2.2. Case 2

A 16-month-old castrated male Maine Coon cat was presented with pelvic limb lameness. The cat was kept exclusively indoors and had undergone elective neutering at another veterinary clinic at 10 months of age, when it weighed 5.1 kg. No history of significant trauma was reported by the owner. At the time of presentation, its body weight was 7.4 kg.

At the time of clinical presentation, the cat had a body condition score (BCS) of 6/9 [13]. Orthopedic examination revealed grade 1 pelvic limb lameness [14], with joint laxity and pain elicited during palpation and manipulation of the affected coxofemoral joint, but no detectable crepitus. Based on these clinical and orthopedic findings, a lesion involving the coxofemoral joint was suspected.

Radiographic examination of the pelvis was performed in a ventrodorsal projection under sedation with medetomidine (0.02 mg/kg; Domitor^®^, Orion Corporation, Espoo, Finland), butorphanol (0.2 mg/kg; Butomidor^®^, Richter Pharma AG, Wels, Austria), and ketamine (3 mg/kg; Narkamon^®^, Bioveta a.s., Ivanovice na Hané, Czech Republic), with the cat positioned in dorsal recumbency and both pelvic limbs extended symmetrically. Radiographic examination revealed abnormalities involving the right proximal femoral physis, raising suspicion of femoral capital physeal separation consistent with slipped capital femoral epiphysis (SCFE) (Figure 2a). Computed tomography (CT) of the pelvis was subsequently performed using a 64-slice multidetector CT scanner (SOMATOM Definition AS 64, Siemens Healthineers, Erlangen, Germany), with a slice thickness of 1 mm and images reconstructed using a high-resolution bone reconstruction kernel. Multiplanar reconstructions were obtained to further characterize the proximal femoral abnormalities. CT findings demonstrated separation of the right femoral capital epiphysis from the femoral neck, consistent with SCFE (Figure 2b). No radiographic or CT abnormalities suggestive of physeal separation were identified in the left (contralateral) proximal femur (Figure 2b). Based on the clinical and imaging findings, right unilateral femoral head and neck excision (FHNE) was recommended as a surgical treatment option.

### 2.3. Surgical Procedure

#### 2.3.1. Anesthesia

Prior to surgery, the cats were premedicated intramuscularly with 0.02 mg/kg medetomidine (Domitor^®^, Orion Corporation, Espoo, Finland), butorphanol 0.2 mg/kg (Butomidor^®^, Richter Pharma AG, Wels, Austria), and 3 mg/kg ketamine (Narkamon^®^, Bioveta a.s., Ivanovice na Hané, Czech Republic). Following placement of an intravenous catheter, general anesthesia was induced with 2 mg/kg alfaxalone i.v. (Alfaxan^®^, Jurox Pty Ltd., Rutherford, NSW, Australia). After orotracheal intubation, anesthesia was maintained with isoflurane in oxygen. Heart rate, respiratory rate, peripheral oxygen saturation (SpO_2_), non-invasive arterial blood pressure, and body temperature were monitored throughout the procedure.

For femoral head and neck excision, each cat was positioned in lateral recumbency with the affected pelvic limb uppermost and the contralateral limb in contact with the operating table. The affected limb and coxofemoral region were clipped and aseptically prepared for surgery.

#### 2.3.2. Surgery

In both cases, FHNE was selected as the surgical treatment primarily due to financial constraints reported by the owners, which limited the feasibility of alternative surgical options, including total hip replacement. In Case 1, bilateral FHNE was performed during a single anesthetic episode, with both coxofemoral joints treated sequentially during the same surgical intervention.

Femoral head and neck excision (FHNE) was performed using a craniolateral approach to the coxo-femoral joint [15]. Following exposure of the joint and incision of the joint capsule, the ligament of the femoral head (ligamentum teres) was identified and transected, allowing removal of the femoral head from the acetabulum. The remaining femoral neck was subsequently resected by osteotomy. The osteotomy surface was carefully inspected and smoothed to eliminate any residual bony prominences that could result in postoperative impingement. The surgical site was thoroughly lavaged with sterile saline solution. The joint capsule was closed with 2-0 polydioxanone (PDO) suture material. The subcutaneous tissue was closed with 3-0 polyglactin 910 (Vicryl^®^), followed by skin closure with 3-0 monofilament polypropylene suture material and a reverse-cutting needle in a simple interrupted pattern. Skin sutures were removed 10 days postoperatively.

Postoperative analgesia consisted of buprenorphine in doses of 0.01 mg/kg, every 6 h (Bupaq^®^, VetViva Richter GmbH, Wels, Austria) administered for three days during hospitalization. Following discharge, both cats received oral robenacoxib in doses of 1 mg/kg every 24 h (Onsior^®^, Elanco GmbH, Cuxhaven, Germany) for five consecutive days as part of their postoperative pain management.

Histopathological examination of the excised femoral heads and necks was not performed in either case, as the owners declined additional diagnostic investigations due to financial constraints, and clinical management was primarily focused on surgical treatment.

#### 2.3.3. Rehabilitation

Postoperative rehabilitation was initiated two weeks after surgery in both cats and included gentle passive flexion and extension exercises of the operated pelvic limbs, with particular attention to the coxofemoral joints. These exercises were performed within a comfortable range of motion to avoid pain or discomfort. Rehabilitation also involved encouraging voluntary walking and progressive weight-bearing on the operated limbs. Owners were instructed to continue gentle mobilization exercises and encourage controlled physical activity at home, while restricting jumping and strenuous activity during the initial recovery period. These measures were intended to maintain joint mobility, encourage limb use, and support progressive muscular recovery.

During postoperative follow-up, both cats showed improvement in pelvic limb function, with resolution of clinically apparent lameness and restoration of weight-bearing on the operated limbs. These improvements were observed during postoperative clinical examinations and were also reported by the owners based on the cats’ mobility and limb use at home.

## 3. Discussion

Slipped capital femoral epiphysis (SCFE), also referred to as spontaneous femoral capital physeal fracture, is an uncommon orthopedic disorder characterized by displacement or separation of the femoral capital epiphysis through the proximal femoral physis in the absence of significant trauma [1,3,15,16]. Several predisposing factors have been proposed, including male sex, neutering, increased body weight, delayed physeal closure, and breed-related characteristics [1,17]. The two cases described in the present report shared several of these features: both were neutered male Maine Coon cats kept exclusively indoors, with no known history of significant trauma. Case 1 was neutered at 8 months and diagnosed with SCFE at 21 months of age, whereas Case 2 was neutered at 10 months and diagnosed at 16 months. Clinically, the first cat exhibited a history of gradually reduced activity followed by grade 2 pelvic limb lameness, bilateral pelvic limb muscle atrophy, and pain and crepitus during manipulation of both coxofemoral joints. In contrast, the second cat presented with grade 1 pelvic limb lameness, joint laxity, and pain without palpable crepitus. Although both cats were diagnosed at a relatively young age, the precise timing of physeal separation could not be established from the available clinical histories.

The body weights recorded in the present cases (7.8 and 7.4 kg, respectively) were comparable to those reported by Borak et al., who documented a mean body weight of 7.5 kg in 17 Maine Coon cats with SCFE [3]. However, both cats in the present report had body condition scores above the ideal range (7/9 and 6/9, respectively), compared with a mean BCS of 5.06/9 in the previously published series [14]. Although increased body condition may contribute to mechanical loading of the proximal femoral physis, its role in the development of SCFE remains uncertain [3,14].

Maine Coon cats appear to be particularly predisposed to SCFE. Previous studies have reported an increased occurrence of this condition in the breed, with male cats being predominantly affected [3]. One possible contributing factor is the relatively prolonged period of skeletal maturation observed in Maine Coons [3]. Delayed physeal closure has been reported in affected Maine Coon cats, with growth plates remaining radiographically open beyond the age at which physeal closure would normally be expected in the general feline population [18]. Persistence of an open physis increases the period during which the growth plate remains exposed to mechanical loading and may therefore represent a site of structural weakness [19].

This mechanism may be particularly relevant to the present cases, in which SCFE was diagnosed at 21 and 16 months of age, respectively, although the precise timing of physeal separation remains unknown. Both cats were kept exclusively indoors and no significant traumatic event was reported. Although minor or unobserved trauma cannot be excluded, persistence or delayed closure of the proximal femoral physis may have contributed to increased mechanical susceptibility at the time of fracture [3,18]. As mechanical stress is repeatedly applied to an abnormal or persistent physis during normal activity, progressive weakening may eventually result in displacement or complete separation of the femoral capital epiphysis [1,19].

The potential influence of neutering on physeal maturation should also be considered. Gonadal hormones are involved in skeletal maturation, and gonadectomy has been associated with delayed closure of some growth plates in cats [19]. However, a direct effect of neutering on closure of the proximal femoral physis has not been clearly demonstrated. Therefore, although both cats in the present report were neutered before fracture diagnosis, this temporal relationship should not be interpreted as evidence of a direct causal association. Breed-related skeletal maturation, body weight, individual variation in physeal closure, and mechanical loading may also contribute to the development of SCFE [18,19].

Clinical presentation of SCFE can vary from progressive or intermittent pelvic limb lameness to an acute onset of marked lameness [20]. This variability was also observed in the present cases. Case 1 presented with grade 2 right pelvic limb lameness, palpable crepitus, and pain during manipulation of the affected coxofemoral joint. In contrast, Case 2 exhibited grade 1 lameness without palpable crepitus, although joint laxity and pain were evident during orthopedic examination. These findings emphasize that relatively mild clinical signs do not exclude a clinically relevant proximal femoral physeal lesion and that careful examination of the coxofemoral joints is warranted in young Maine Coon cats presenting with unexplained pelvic limb lameness.

Muscle atrophy was also evident in both cases and may represent an additional indicator of the chronicity of the underlying coxofemoral disease. In Case 1, reduced soft-tissue volume consistent with muscle atrophy was observed bilaterally, whereas in Case 2, muscle atrophy was evident in the affected pelvic limb. These changes may reflect prolonged reduction in limb use associated with pain, altered weight-bearing, and decreased physical activity. In cats with orthopedic disease, persistent lameness and reduced use of the affected limb may contribute to progressive loss of muscle mass [16,18]. In the present cases, the presence of muscle atrophy, together with the owners’ reports of reduced physical activity and the imaging evidence of chronic changes, supports a prolonged course of the coxofemoral pathology rather than a purely acute traumatic event. Nevertheless, muscle atrophy is a nonspecific finding and should be interpreted in conjunction with the clinical history, orthopedic examination, and diagnostic imaging findings.

Diagnostic imaging is essential for confirming SCFE. Conventional radiography remains the first-line imaging modality and may reveal physeal widening or irregularity, displacement of the femoral capital epiphysis, femoral neck osteolysis or sclerosis, and metaphyseal remodeling [11,18]. In Case 1, radiographic examination was sufficient to establish the diagnosis. In Case 2, CT was performed with the pelvic limbs positioned in a frog-leg configuration and provided detailed cross-sectional assessment of the proximal femoral lesion. CT eliminated anatomical superimposition and allowed detailed characterization of the physeal separation, epiphyseal displacement, and associated changes in the femoral neck. This imaging modality may therefore be particularly useful when a more detailed anatomical assessment of the proximal femur is required for diagnosis or surgical planning [16,19]. In the present cases, the clinical history, absence of significant trauma, and diagnostic imaging findings were consistent with SCFE. However, histopathological examination of the excised femoral heads and necks was not performed. Although histopathological abnormalities of the proximal femoral physis have been described in cats with SCFE [3,6], the absence of histological examination in the present cases prevented confirmation of underlying physeal dysplasia. Therefore, the proposed involvement of physeal dysplasia remains speculative, and the diagnosis should be interpreted in the context of the available clinical and imaging findings.

Femoral head and neck excision (FHNE) was selected as the surgical treatment in both cats. This procedure eliminates painful contact between the proximal femur and acetabulum and allows subsequent formation of a functional fibrous pseudoarthrosis [17,20]. Careful surgical technique and preservation of the surrounding soft tissues are also important to facilitate postoperative recovery [16]. Primary surgical stabilization may represent an alternative to salvage procedures in selected cases of femoral capital physeal fracture when adequate reduction and stable fixation can be achieved. However, chronicity of the lesion and reduced bone stock may preclude successful primary stabilization, making FHNE a more appropriate option [21]. Therefore, the choice of surgical technique should be based on lesion chronicity and configuration, the degree of epiphyseal displacement, available bone stock, and the feasibility of achieving stable anatomical reduction [22].

Bilateral involvement represents an additional consideration in the management of feline SCFE, as physeal abnormalities may affect both proximal femora. In the present report, one cat exhibited bilateral femoral capital physeal separation associated with secondary degenerative changes and underwent bilateral FHNE. When clinically relevant lesions are present bilaterally, bilateral FHNE may represent an appropriate salvage option, particularly when chronic changes or extensive physeal separation limit the feasibility of anatomical reduction and internal fixation. Previous reports have described bilateral SCFE in cats and have shown that bilateral FHNE can be performed either during a single anesthetic episode or as staged procedures, depending on the severity of the lesions and the clinical status of the patient [23,24]. Despite the more extensive nature of bilateral surgery, cats generally have a favorable capacity for functional adaptation following FHNE, although adequate postoperative analgesia and early controlled limb use remain important for recovery [24].

The persistence of the third trochanter following FHNE, as observed in Case 2, should also be considered when evaluating postoperative outcomes. Although residual bony prominences may potentially contribute to mechanical impingement and persistent lameness [3,16], no clinically apparent lameness was observed during follow-up in the present case. The persistence of pelvic limb muscle atrophy at six months postoperatively suggests that muscular recovery may have remained incomplete despite the observed improvement in limb use and resolution of clinically apparent lameness.

The main limitations of this retrospective case report are the small number of cases and the absence of standardized postoperative functional assessments. Although clinical improvement, including resolution of lameness and restoration of pelvic limb use, was observed, objective gait analysis and structured owner-reported assessments were not performed. Furthermore, postoperative rehabilitation was not standardized, and its effectiveness was not objectively evaluated. The different diagnostic imaging approaches used in the two cases also limit direct comparison of the findings. In addition, serum gonadal hormone concentrations were not assessed, preventing evaluation of the endocrine status of the cats and its potential relationship with physeal maturation. Histopathological examination of the excised femoral heads and necks was not performed due to financial constraints. Consequently, underlying physeal dysplasia could not be confirmed microscopically. Although both cats were neutered before SCFE diagnosis and no significant traumatic event was reported, a causal relationship between neutering and physeal failure cannot be established. Minor unobserved trauma and other contributing factors cannot be excluded. Finally, as SCFE in Maine Coon cats has been previously documented, the present cases provide additional clinical observations rather than a novel disease presentation. Further studies involving larger populations, standardized functional assessments, and endocrine and histopathological investigations are warranted.

## 4. Conclusions

These cases highlight the importance of considering slipped capital femoral epiphysis (SCFE) in the differential diagnosis of pelvic limb lameness in young male Maine Coon cats, particularly in the absence of significant trauma. Although both cats had been neutered before diagnosis, a causal relationship between neutering and physeal failure cannot be established. Following femoral head and neck excision (FHNE), both cats showed clinical improvement, including resolution of clinically apparent lameness and restoration of pelvic limb use, as observed during postoperative examinations and reported by the owners. However, persistent muscle atrophy at the six-month follow-up and the absence of standardized functional assessments preclude definitive conclusions regarding the extent of functional recovery and the effectiveness of FHNE.

## Figures and Tables

**Figure 1:**
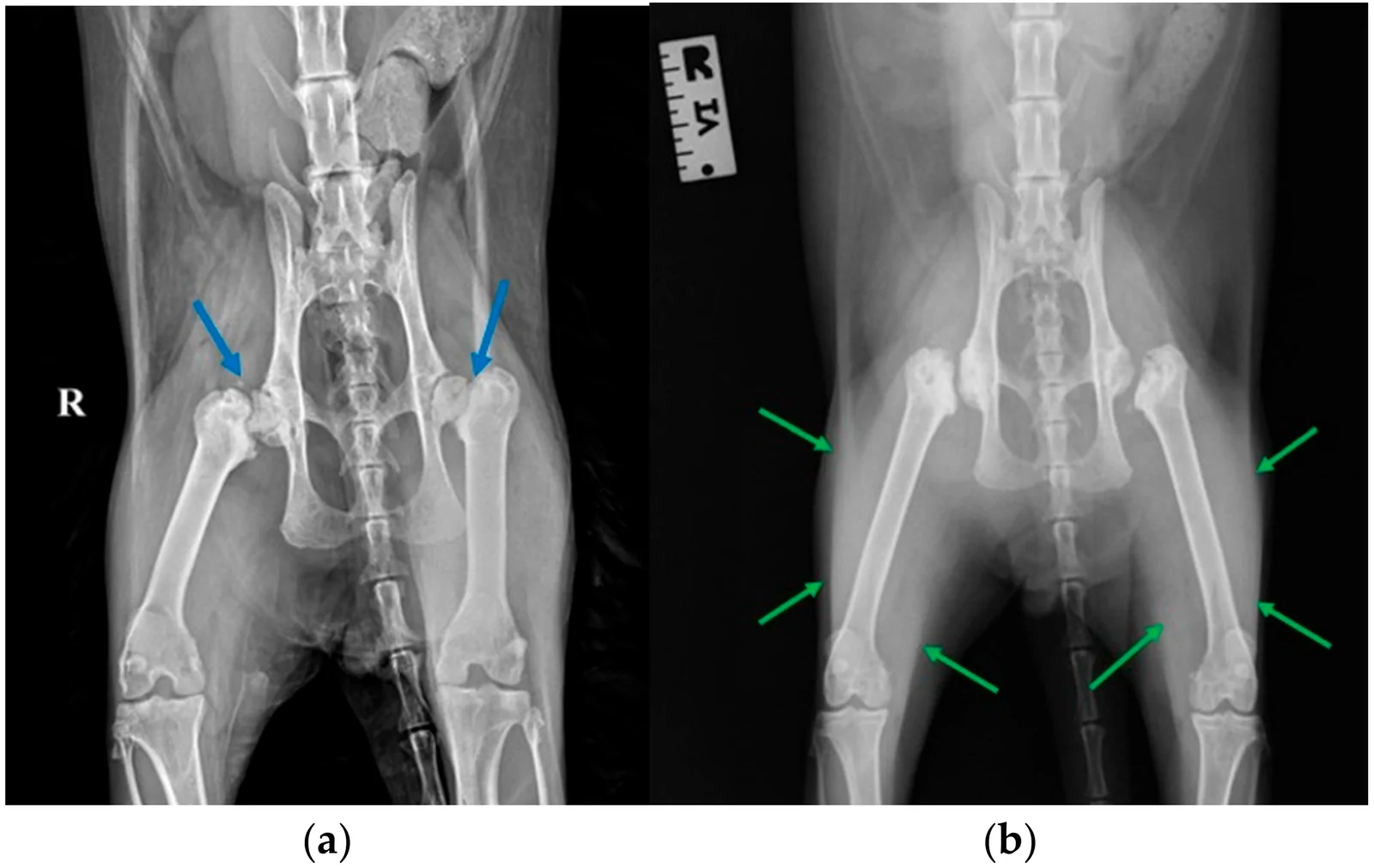
Preoperative and follow-up ventro-dorsal radiographic views of the pelvis. (**a**) Preoperative radiograph showing bilateral separation and displacement of the femoral capital epiphyses (blue arrows), consistent with slipped capital femoral epiphysis (SCFE), with more pronounced changes involving the right proximal femur. (**b**) Six-month postoperative ventrodorsal radiograph following bilateral femoral head and neck excision (FHNE), demonstrating removal of the femoral heads and necks without apparent osseous contact between the proximal femora and the acetabula. Green arrows indicate bilateral reduction in pelvic limb soft-tissue volume, consistent with muscle atrophy.

**Figure 2:**
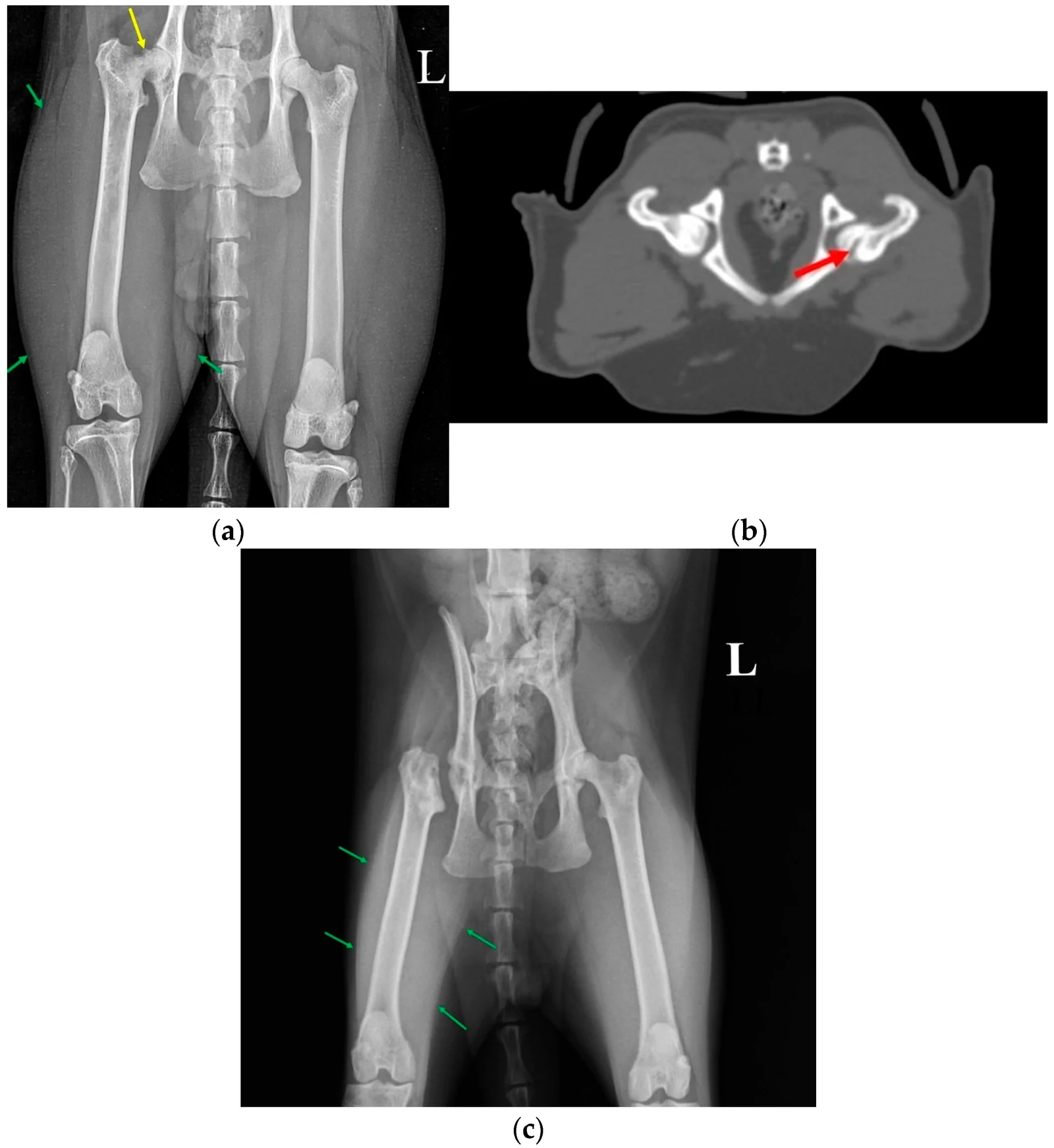
Preoperative and postoperative diagnostic imaging of Case 2. (**a**) Preoperative ventrodorsal radiographic view of the pelvis showing separation and displacement of the right femoral capital epiphysis through the proximal femoral physis, consistent with slipped capital femoral epiphysis (SCFE) (yellow arrow). Green arrows indicate reduced soft-tissue volume of the right pelvic limb, consistent with muscle atrophy. (**b**) Computed tomography image of the pelvis demonstrating separation of the right femoral capital epiphysis through the proximal femoral physis (red arrow), consistent with SCFE. (**c**) Six-month postoperative ventrodorsal radiographic view following right femoral head and neck excision (FHNE), with green arrows indicating reduced soft-tissue volume of the affected pelvic limb, consistent with muscle atrophy.

## Data Availability

The original contributions presented in this study are included in the article. Further inquiries can be directed to the corresponding author(s).

## Abbreviations

The following abbreviations are used in this manuscript:

SCFE	Slipped capital femoral epiphysis
FHNE	Femoral head and neck excision
CT	Computed tomography
PDO	Polydioxanone
